# Sample Size for Oxidative Stress and Inflammation When Treating Multiple Sclerosis with Interferon-β1a and Coenzyme Q10

**DOI:** 10.3390/brainsci9100259

**Published:** 2019-09-27

**Authors:** Marcello Moccia, Antonio Capacchione, Roberta Lanzillo, Fortunata Carbone, Teresa Micillo, Giuseppe Matarese, Raffaele Palladino, Vincenzo Brescia Morra

**Affiliations:** 1 Multiple Sclerosis Clinical Care and Research Centre, Department of Neuroscience, Reproductive Science and Odontostomatology, Federico II University, 80131 Naples, Italy; robertalanzillo@libero.it (R.L.); vincenzo.bresciamorra2@unina.it (V.B.M.); 2 Medical Affairs Department, Merck, 00176 Rome, Italy; antonio.capacchione@merckgroup.com; 3 Neuroimmunology Unit, IRCCS Fondazione Santa Lucia, 00142 Rome, Italy; fortunata.carbone@alice.it; 4 Laboratory of Immunology, Institute of Experimental Endocrinology and Oncology, National Research Council (IEOS-CNR), 80131 Naples, Italy; giuseppe.matarese@unina.it; 5 Department of Biology, Federico II University, 80131 Naples, Italy; teresa.micillo2@unina.it; 6 Treg Cell Lab, Department of Molecular Medicine and Medical Biotechnologies, Federico II University, 80131 Naples, Italy; 7 Department of Primary Care and Public Health, Imperial College, London W68RP, UK; palladino.raffaele@gmail.com; 8 Department of Public Health, Federico II University, 80131 Naples, Italy

**Keywords:** multiple sclerosis, inflammation, oxidative, biomarker, sample size

## Abstract

Studying multiple sclerosis (MS) and its treatments requires the use of biomarkers for underlying pathological mechanisms. We aim to estimate the required sample size for detecting variations of biomarkers of inflammation and oxidative stress. This is a post-hoc analysis on 60 relapsing-remitting MS patients treated with Interferon-β1a and Coenzyme Q10 for 3 months in an open-label crossover design over 6 months. At baseline and at the 3 and 6-month visits, we measured markers of scavenging activity, oxidative damage, and inflammation in the peripheral blood (180 measurements). Variations of laboratory measures (treatment effect) were estimated using mixed-effect linear regression models (including age, gender, disease duration, baseline expanded disability status scale (EDSS), and the duration of Interferon-β1a treatment as covariates; creatinine was also included for uric acid analyses), and were used for sample size calculations. Hypothesizing a clinical trial aiming to detect a 70% effect in 3 months (power = 80% alpha-error = 5%), the sample size per treatment arm would be 1 for interleukin (IL)-3 and IL-5, 4 for IL-7 and IL-2R, 6 for IL-13, 14 for IL-6, 22 for IL-8, 23 for IL-4, 25 for activation-normal T cell expressed and secreted (RANTES), 26 for tumor necrosis factor (TNF)-α, 27 for IL-1β, and 29 for uric acid. Peripheral biomarkers of oxidative stress and inflammation could be used in proof-of-concept studies to quickly screen the mechanisms of action of MS treatments.

## 1. Introduction

Monitoring multiple sclerosis (MS) and developing new disease modifying treatments (DMTs) requires the use of biomarkers for underlying pathological mechanisms [1,2]. Thus, it is crucial to define a set of biomarkers that can be easily measured (e.g., in accessible body fluids), are quickly responsive to change, and reflect MS clinical features accurately [2,3].

Experimental evidence supports the important role of inflammation and oxidative stress in the pathogenesis of MS [4]. In the initial relapsing-remitting (RR) phase, oxidative stress is strictly associated with inflammatory activity, whereas the progressive phase is characterized by chronic inflammation and neurodegeneration, further amplifying the oxidative damage [4,5]. In our recent study [6], supplementation with Coenzyme Q10, a natural anti-oxidant, along with Interferon-β1a 44 mcg treatment, was associated with an improved oxidative balance, with a shift toward an anti-inflammatory milieu and with related clinical benefits. However, in this study we used a large number of peripheral biomarkers of oxidative stress and inflammation, which was time-and resource-consuming, and ultimately resulted in a significant statistical challenge due to multiple comparisons [6]. Thus, future studies would benefit from a subset of biomarkers that are sensitive to change in a short time and on a small sample.

In the present post-hoc analysis of our previous longitudinal study, we aim to estimate the sample size needed in RR-MS for different peripheral biomarkers of oxidative stress and inflammation.

## 2. Materials and Methods

### 2.1. Study Design and Population

This is a post-hoc analysis on a prospective cohort that was fully described elsewhere [6]. Briefly, in 2016–2017, we included 60 RRMS patients on clinical stability and on treatment with subcutaneous high-dose Interferon-β1a (Rebif^®^, 44 mcg, Merck, Rome, Italy), either alone or with Coenzyme Q10 (Skatto^®^, 100 mg/ml, Chiesi Farmaceutici SpA, Parma, Italy) for 3 months, with a cross-over design. In particular, group 1 (*n* = 30) was treated with Interferon-β1a and Coenzyme Q10 from baseline to a 3-month visit, and then with Interferon-β1a alone until a 6-month visit; meanwhile, group 2 (*n* = 30) was treated with Interferon-β1a alone from baseline to a 3-month visit, and then with Interferon-β1a and Coenzyme Q10 until a 6-month visit. This design used within-subjects comparison of treatments, and therefore minimized confounding variables by removing any natural biological variation that may have occurred in the measurement of the outcome measures [6,7].

### 2.2. Laboratory Analyses

Blood samples were collected at baseline and after 3 and 6 months (60 patients with 3 laboratory measurements, with 180 measurements overall) in fasting conditions in lithium heparin tubes, immediately centrifuged, stored at −80 °C, and then analyzed for:

(1) Markers of free radical scavenging activity: uric acid and bilirubin were measured by using the UA2 and the BILTS enzymatic methods (COBAS^®^ c501 analyser, Roche Diagnostic, Mannheim, Germany);

(2) Markers of serum oxidative damage: 8-hydroxy-2-deoxyguanosine (8-OHdG, an end product of oxidative DNA damage) and protein carbonyls (an end product of oxidative protein damage) were measured by using the OxiSelect™ Oxidative DNA Damage ELISA kit, and the OxiSelect™ Protein Carbonyl ELISA Kit (both from Cell Biolabs, San Diego, CA, USA);

(3) Markers of inflammation: the Human Cytokine Magnetic 35-Plex Panel (Invitrogen by Thermo Fisher Scientific, Waltham, MA, USA) was used for the quantitative detection of epidermal growth factor (EGF), eotaxin, basic-fibroblast growth factor (FGF), granulocyte-colony stimulating factor (G-CSF), granulocyte-macrophage colony-stimulating factor (GM-CSF), hepatocyte growth factor (HGF), Interferon (IFN)-α, IFN-γ, interleukin (IL)-1α, IL-1β, IL-1RA, IL-2, IL-2R, IL-3, IL-4, IL-5, IL-6, IL-7, IL-8, IL-9, IL-10, IL-12, IL-13, IL-15, IL-17A, IL-17F, IL-22, IFN-γ-inducible protein (IP)-10, monocyte chemoattractant protein (MCP)-1, monokine induced by IFN-γ (MIG), macrophage inflammatory proteins (MIP)-1α, MIP-1β, regulated on activation-normal T cell expressed and secreted (RANTES), tumor necrosis factor (TNF)-α, and vascular endothelial growth factor (VEGF).

CellROX^®^ Orange Reagent (Life Technologies, Carlsbad, CA, USA) was used for measuring intracellular reactive oxygen species (ROS) production in peripheral blood mononuclear cells (PBMCs) using a FACScanto II analyzer (Becton–Dickinson, San Diego, CA, USA) and Flow-Jo v10 software (Tree Star Inc., Ashland, OR, USA); intracellular ROS production (CellROX) was measured as percent positive cells (%) and mean fluorescence intensity (MFI).

### 2.3. Statistics

The sample size needed to detect a treatment effect on different markers of oxidative stress and inflammation was computed using the formula $n=\frac{2{\left(Z_{\alpha }+Z_{1-\beta }\right)}^{2}\sigma^{2}}{\Delta^{2}}$, where *n* is the required sample size per treatment arm in 1:1 controlled trials, *Z_α_* and *Z_1-β_* are constants (set at 5% alpha-error and 80% power, respectively), *σ* is the standard deviation, and *Δ* the estimated effect size [8,9]. The treatment effect was defined as the actual observed effect in our previous study (i.e., variation in each laboratory measure between treated and untreated groups), estimated using mixed-effect linear regression models (including age, gender, disease duration, baseline expanded disability status scale (EDSS), and duration of Interferon-β1a treatment prior to study inclusion as covariates; creatinine was also included for uric acid analyses) [6,8,9]. The crossover model included random effects for patient ID, and fixed-effects for time (baseline, 3 and 6 months), and for the visit after Coenzyme Q10 exposure, overall accounting for possible carry-over effects. Adjusted beta-coefficients of 3-month variations were obtained for each laboratory measure. We assumed that the observed variation, as estimated by the adjusted beta-coefficients, was the highest achievable treatment effect (100%) over 3 months. From there, with a conservative approach, we hypothesized a number of effect sizes—e.g., 30%, 50%, 70%, and 90%—that were smaller than the observed effect. Standard deviations were calculated from the variation of each laboratory measure after 3 months. Then, we hypothesized a clinical trial where two different biomarkers were included as primary outcome measures for sample size estimates (alpha-error was set at 2.5%). Finally, we considered that the study was designed to include one or two interim analyses in addition to the final analysis (alpha-error was set at 2.94% and 2.21%, respectively, according to the Pocock method) [10,11].

Stata 15.0 (StataCorp LLC, College Station, TX, USA) was used for data processing and analysis.

## 3. Results

Sixty RRMS patients were included in the present study (age: 41.5 ± 9.7 years; female: *n* = 42 (70%); disease duration: 11.0 ± 1.7 years; baseline EDSS: 2.5 (1.0–5.0)). Four patients presented with a clinical relapse (6.6%) during the study period.

**Figure 1:**
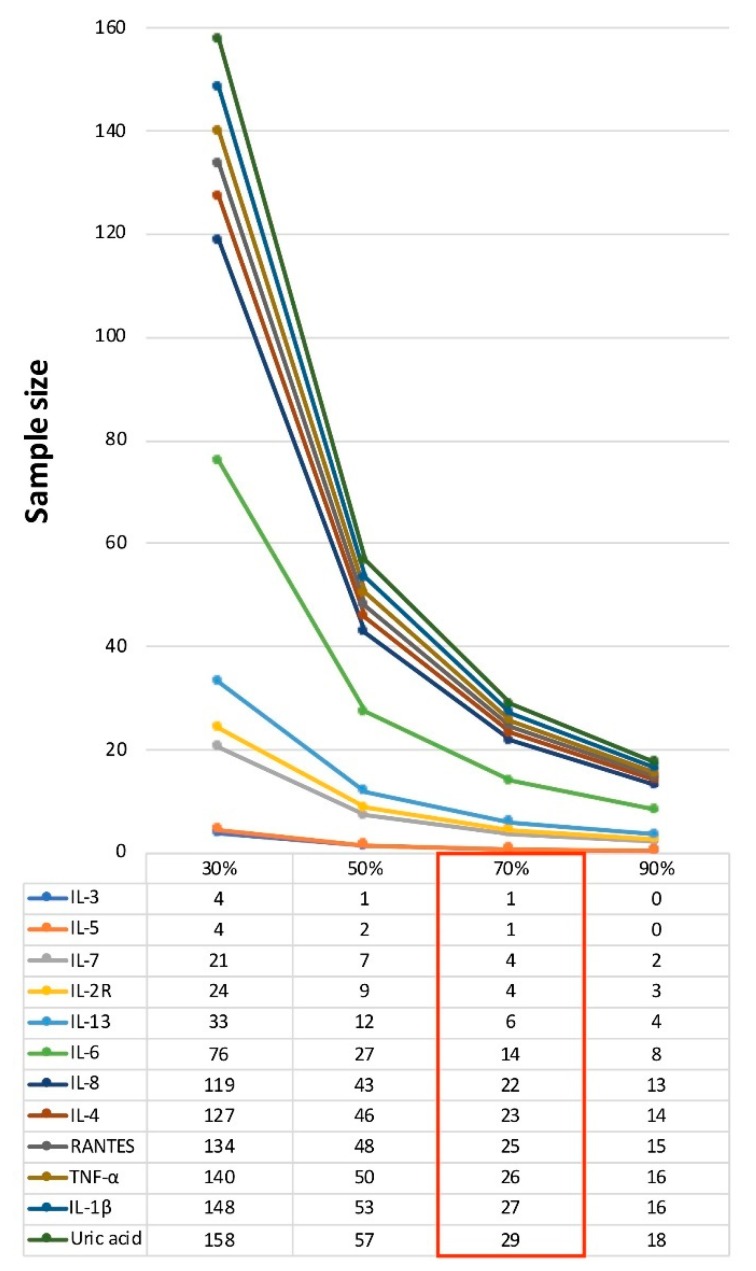
Profile plot for sample size estimates for a treatment arm. Figure shows sample sizes for laboratory markers of oxidative stress and inflammation (<30 patients for a treatment arm with a 70% treatment effect). Sample size per treatment arm is reported hypothesizing a 30%, 50%, 70%, and 90% treatment effect compared with the observed effect. Power was set at 80% and alpha-error at 5%. Abbreviations: interleukin (IL), regulated on activation-normal T cell expressed and secreted (RANTES), and tumor necrosis factor (TNF).

Hypothesizing a clinical trial aiming to detect 70% effect in 3 months (power = 80% alpha-error = 5%), the sample size per treatment arm would be 1 for IL-3 and IL-5, 4 for IL-7 and IL-2R, 6 for IL-13, 14 for IL-6, 22 for IL-8, 23 for IL-4, 25 for RANTES, 26 for TNF-α, 27 for IL-1β, and 29 for uric acid (Figure 1, Table 1). Other investigated markers presented with a sample size per treatment arm larger than 30 (Table 1).

Hypothesizing the combination of two different biomarkers as primary outcome measures (alpha-error = 2.5%), sample size estimates per treatment arm remained substantially favorable (3 for IL-3 and IL-5, 7 for IL-7, 8 for IL-2R, 9 for IL-13, 19 for IL-6, 28 for IL-8, 30 for IL-4, 32 for RANTES, 33 for TNF-α, 35 for IL-1β, and 37 for uric acid) (Table 1).

Sample size estimates for a study with one or two interim analyses (Pocock method, setting alpha-error = 2.94% and 2.21% respectively), in addition to the final analysis, are presented in Table 1; this design would reduce study participants’ exposure to an inferior or useless treatment.

## 4. Discussion

Peripheral biomarkers of inflammation, scavenging activity, and oxidative damage gave realistically achievable sample size estimates, and could be used in exploratory clinical trials and observational studies to screen new or already existing medications with putative effects on inflammation and oxidative stress over a 3-month period. Not least, interim analyses could detect an inferior or useless treatment even earlier, with subsequent study termination or treatment switch within adaptive designs [12].

Current sample size calculations were rather conservative. In particular, in the Results (Section 3) and in Figure 1, we specifically focused on a 70% treatment effect, which was smaller than what we actually observed (100% treatment effect) [6,9]. However, greater treatment effects could be hypothesized with different medications and doses, leading to even smaller sample size estimates. Also, the inclusion of multiple markers as primary outcome measures would remain feasible for sample size calculations. Of note, present estimates are based on the combination of subcutaneous high-dose Interferon-β1a (Rebif^®^, 44 mcg, Merck, Rome, Italy) and Coenzyme Q10. For a subgroup of patients (50%), the Interferon-β1a treatment was also administered prior to study inclusion. Drug naïve patients were equally distributed between Coenzyme Q10 treatment groups, and we also included the duration of the Interferon-β1a treatment as a covariate in the statistical models, but, of course, we cannot exclude the possibility that previous treatment has affected the study outcomes. However, if we assume Interferon-β1a could have exerted its effects before inclusion in the study, we would have observed smaller Coenzyme Q10-related effects, resulting in subsequently more conservative sample size estimates. Interferon-β1a is an approved treatment for MS, with a well-established long-term efficacy and safety profile [13]. On the contrary, Coenzyme Q10 has proven effect on biomarkers of oxidative stress and inflammation and on MS symptoms [14,15,16], but its disease-modifying effect remains to be established. As such, future studies should evaluate the reproducibility of our findings on more recent medications (e.g., cladribine).

Most promising inflammatory biomarkers are strongly related to MS pathogenesis, and in particular, to acute (e.g., IL-1β, IL-3) and chronic inflammation (e.g., IL-2R, IL-6, IL-7, IL-8, TNF-α) within the central nervous system [17,18,19,20,21], to suppression of the activity of microglia toward brain repair (i.e., RANTES), and to neuroprotective modulation of pathologically-active macrophages and microglia (e.g., IL-4, IL-13) [17,22]. Markers of oxidative stress also resulted in rather small sample sizes, with particular regard to markers of serum scavenging activity (uric acid), and of oxidative damage in inflammatory cells and DNA (CellROX, %, and 8-OHdG). Biomarkers of oxidative stress and inflammation are not only related to MS pathogenesis [17,23], but are also clinically relevant to MS, being associated with MS risk and progression [6,17,21,24,25,26], and also being used as therapeutic targets [17]. For instance, IL-6, IL-8, and RANTES have been associated with the risk of clinical relapses [27], radiological activity (e.g., lesions, atrophy) [28], treatment switch, and disability progression after up to 6 years [20]. Interestingly, clinical associations might be particularly sound in patients in apparent clinical stability [29]. As such, longitudinal measurements of oxidative stress and inflammation can provide pathologically and clinically relevant information in MS observational studies and clinical trials.

Of note, for some inflammatory biomarkers (e.g., IL-3 and IL-5) sample size estimates were unexpectedly low and should be interpreted with caution. If we assume we are studying a compound with a specific molecular target (e.g., anti-TNF-α or anti-CD20 antibodies), then only a very small sample is necessary to detect biological effect [30,31]. On the contrary, for compounds with multimodal mechanisms of action, a larger sample would be needed or, at least, profiles of inflammatory pathology should be considered [26].

Limitations of this study include possible confounding factors. In our previous study, we excluded patients with possible confounding factors (e.g., contraceptive and immunosuppressive medication), we used within-patients comparison of treatments (minimizing confounding effects by removing any natural biological variation), and we accounted for a number of covariates in our statistical models [6], but factors influencing oxidative stress and inflammation are multiple and virtually impossible to exclude completely. For instance, four patients presented with a clinical relapse (6.6%) that we did not account for considering that patients were equally distributed in the Coenzyme-Q10-treated and untreated groups. Specificity of peripheral biomarkers to MS-related pathology remains to be further investigated, and based on current knowledge, these markers cannot replace conventional biomarkers of disability (e.g., neuroimaging) [32]. We included 180 measurements at three timepoints from 60 patients to estimate coefficients of variation for sample estimates. As such, included sample could have been larger, but was based on sample size calculations from our previous study, and not least, was in line with previous studies with similar goals [6,8,33]. Also, measurements over short intervals may be prone to increased measurement errors leading to a greater variability and larger sample, but apparently, this was not the case in our cohort. A control group (untreated or treated with a medication different from Interferon-β1a) was unfortunately not available, with difficulties in drawing formal conclusions on the observed effects.

## 5. Conclusions

In conclusion, peripheral biomarkers of oxidative stress and inflammation could be used in exploratory, proof-of-concept studies aiming to evaluate the activity profile of new or already existing medications. Medications with putative anti-oxidant and anti-inflammatory effects could be tested in a short time (3 months) and on small samples (<30 per treatment arm) by using a limited subset of biomarkers, before being moved toward larger and more expensive clinical trials.

## Figures and Tables

**Table 1:** Sample size estimates for a treatment arm for 3-month variations of peripheral biomarkers of oxidative stress and inflammation.

	Baseline	Adj. Coeff. (3-Month Variation)	SD (3-Month Variation)	Sample Size (70% Treatment Effect)			
				One Primary Outcome	Two Primary Outcomes	Interim Analyses (Pocock Method)	
						One Interim	Two Interim
				5% alpha	2.5% alpha	2.94% alpha	2.21% alpha
Markers of scavenging activity							
Uric acid (mg/dL)	4.670 ± 0.566	0.123 *	0.117	29	37	15	11
Bilirubin (mg/dL)	1.466 ± 0.268	0.066	0.190	265	323	134	98
Markers of oxidative damage							
CellROX cells (%)	76.405 ± 9.348	−9.925 *	11.25	41	52	21	15
CellROX cells (MFI)	2605.320 ± 828.707	−523.308 *	1124.538	148	181	75	55
Protein carbonyls (nmol/mg)	2.976 ± 1.402	−0.266	1.393	878	1066	444	326
8-OHdG (ng/mL)	6.379 ± 1.140	−0.630 *	0.708	40	51	20	15
Markers of inflammation							
EGF (pg/mL)	6.597 ± 12.877	−3.637	8.513	175	214	89	65
Eotaxin (pg/mL)	116.432 ± 46.800	−18.669 *	31.968	94	116	47	35
Basic-FGF (pg/mL)	53.218 ± 282.165	−2.736	4.863	101	124	51	38
G-CSF (pg/mL)	80.445 ± 48.370	−4.692	61.503	5498	6667	2783	2041
GM-CSF (pg/mL)	5.791 ± 4.953	−1.751 *	2.524	66	82	34	25
HGF (pg/mL)	64.959 ± 77.650	−26.397 *	33.925	53	66	27	20
IFN-α (pg/mL)	80.869 ± 469.445	1.780	11.498	1335	1618	676	496
IFN-γ (pg/mL)	2.311 ± 1.952	−1.526 *	1.937	52	64	26	19
IL-1α (pg/mL)	4.427 ± 6.477	−2.460 *	2.526	34	43	17	13
IL-1β (pg/mL)	1.694 ± 7.274	−1.188	1.096	27	35	14	10
IL-1RA (pg/mL)	33.085 ± 39.824	−10.464	18.329	98	121	50	36
IL-2 (pg/mL)	20.979 ± 107.943	5.099	14.090	244	298	124	91
IL-2R (pg/mL)	105.950 ± 61.462	−29.971 *	11.182	4	8	2	2
IL-3 (pg/mL)	202.849 ± 1283.170	28.661	4.276	1	3	0	0
IL-4 (pg/mL)	4.421 ± 11.691	3.883 *	3.317	23	30	12	9
IL-5 (pg/mL)	8.177 ± 33.861	−12.890	2.069	1	3	0	0
IL-6 (pg/mL)	62.945 ± 363.131	5.559	3.671	14	19	7	5
IL-7 (pg/mL)	12.871 ± 40.625	−16.428	5.639	4	7	2	1
IL-8 (pg/mL)	12.095 ± 7.422	−11.418	9.425	22	28	11	8
IL-9 (pg/mL)	2.248 ± 4.814	−3.749 *	4.212	40	51	20	15
IL-10 (pg/mL)	1079.590 ± 6456.040	1615.546	2417.951	72	89	36	27
IL-12 (pg/mL)	58.932 ± 110.51	2.498	14.365	1058	1284	536	393
IL-13 (pg/mL)	1.714 ± 3.341	3.732 *	1.628	6	9	3	2
IL-15 (pg/mL)	117.149 ± 673.398	21.693	21.658	32	40	16	12
IL-17A (pg/mL)	1.460 ± 2.265	−0.453	0.941	138	169	70	51
IL-17F (pg/mL)	35.954 ± 86.735	−68.854 *	72.039	35	44	18	13
IL-22 (pg/mL)	250.425 ± 642.791	−8.406	40.134	729	886	369	271
IP-10 (pg/mL)	26.279 ± 16.844	5.699	30.460	914	1110	463	339
MCP-1 (pg/mL)	232.083 ± 79.633	39.540	96.247	190	232	96	70
MIG (pg/mL)	32.386 ± 13.580	−5.409	13.555	201	245	102	75
MIP-1α (pg/mL)	7.830 ± 11.718	−5.327 *	5.338	32	41	16	12
MIP-1β (pg/mL)	182.476 ± 1024.490	17.125	17.060	32	40	16	12
RANTES (pg/mL)	1739.970 ± 1475.350	−2331.281 *	2041.081	25	32	12	9
TNF-α (pg/mL)	2.725 ± 4.310	−1.795 *	1.608	26	33	13	10
VEGF (pg/mL)	0.619 ± 0.777	−0.398 *	0.519	54	68	28	20

Table shows absolute values of biomarkers of oxidative stress and inflammation at the baseline visit. Adjusted beta-coefficients (adj. coeff.) of 3-month variation for each laboratory measure were obtained with mixed-effect linear regression models (including age, gender, disease duration, baseline EDSS, and duration of Interferon-β1a treatment prior to study inclusion as covariates; creatinine was also included for uric acid analyses) (* indicates *p* < 0.05). Standard deviation (SD) was calculated from the variation of each laboratory measure after 3 months. Sample size per treatment arm is reported, hypothesizing a 70% treatment effect, compared with the observed effect, over 3 months (power was set at 80%, alpha-error was set at 5%). Then, we also performed calculations hypothesizing additional scenarios: (i) two different biomarkers were included as combined primary outcome measures for sample size estimates (alpha-error was set at 2.5%); (ii) the study was designed to include one or two interim analyses in addition to the final analysis in order to obtain early evidence of inferior or useless treatment (alpha-error was set to be 0.0294 and 0.0221, respectively, according to the Pocock method). Abbreviations: intracellular ROS production (CellROX), mean fluorescence intensity (MFI), 8-hydroxy-2-deoxyguanosine (8-OHdG), epidermal growth factor (EGF), eotaxin, basic-fibroblast growth factor (FGF), granulocyte-colony stimulating factor (G-CSF), granulocyte-macrophage colony-stimulating factor (GM-CSF), hepatocyte growth factor (HGF), interferon (IFN)- α, IFN-γ, interleukin (IL)-1α, IL-1β, IL-1RA, IL-2, IL-2R, IL-3, IL-4, IL-5, IL-6, IL-7, IL-8, IL-9, IL-10, IL-12, IL-13, IL-15, IL-17A, IL-17F, IL-22, IFN-γ-inducible protein (IP)-10, monocyte chemoattractant protein (MCP)-1, monokine induced by IFN-γ (MIG), macrophage inflammatory proteins (MIP)-1α, MIP-1β, regulated on activation-normal T cell expressed and secreted (RANTES), tumor necrosis factor (TNF)-α, and vascular endothelial growth factor (VEGF).
